# Fission yeast metabolome dynamics during phosphate starvation and replenishment

**DOI:** 10.1128/mbio.00241-25

**Published:** 2025-02-25

**Authors:** Ana M. Sanchez, Aye K. Kyaw, Sara Nunes Violante, Angad Garg, Justin R. Cross, Stewart Shuman

**Affiliations:** 1 Molecular Biology Program, Memorial Sloan Kettering Cancer Center, New York, New York, USA; 2 Gerstner Sloan Kettering Graduate School of Biomedical Sciences, New York, New York, USA; 3 Donald B. and Catherine C. Marron Cancer Metabolism Center, MSKCC, New York, New York, USA; Harvard Medical School, Boston, Massachusetts, USA

**Keywords:** phosphate metabolism, phosphate starvation, *Schizosaccharomyces pombe*

## Abstract

Inorganic phosphate is an essential nutrient acquired by cells from their environment and assimilated into myriad intracellular metabolites and macromolecules. Here, we characterize the metabolic responses of fission yeast to a 24 h interval of phosphate starvation, during which cells enter a state of G0 quiescence. Time-resolved profiling revealed that many key phosphometabolites were progressively depleted, including (i) NTPs, NDPs, and dNTPs; (ii) coenzyme A, NAD^+^, NADP^+^, NADH, and ADP-ribose; (iii) glycolysis pathway intermediates upstream of pyruvate; (iv) pentose phosphate pathway intermediates from 6-phosphogluconate to sedoheptulose-7-phosphate; (v) nucleotide sugars GDP-hexose, UDP-glucose/galactose, and UDP-GalNAc/GlcNAc; and (vi) phospholipid precursors glycerol-3-phosphate, CDP-choline, and glycerophosphocholine. By contrast, early Krebs cycle intermediates accumulated during phosphate starvation. Other metabolic changes included the following: (i) interdiction of *de novo* pyrimidine synthesis; (ii) depletion of *S*-adenosylmethionine and *S*-adenosylhomocysteine; (iii) transient accumulation of polyamine biosynthetic intermediates putrescine and 5-methylthioadenosine; (iv) accumulation of betaine (correlating with an increase in expression of *atd1* mRNA encoding aldehyde dehydrogenase); and (v) depletion of aminoadipate pathway intermediates 2-oxoadipate, 2-aminoadipate, and saccharopine. Replenishing phosphate after 24 h of starvation resulted in restoration of the pre-starvation metabolome (over 2 to 12 h) as cells exited quiescence and resumed growth.

**IMPORTANCE:**

Fission yeast *Schizosaccharomyces pombe* is a valuable model system to study cellular phosphate homeostasis and the adaptive responses to chronic phosphate starvation. Previous analyses focused on changes in the fission yeast transcriptome and proteome during phosphate starvation-induced durable G0 quiescence. Here, we deployed metabolomics to survey the scope and temporal order of metabolite changes during 24 h of phosphate starvation and the kinetics of metabolic recovery after cells starved for 24 h are replenished with phosphate. These results contribute to a multi-omics understanding of how phosphate status impacts cell cycle, gene expression, metabolism, and chronological lifespan.

## INTRODUCTION

Inorganic phosphate is an essential nutrient. Phosphate acquired from the extracellular milieu is assimilated by cells into diverse molecules with distinctive bond chemistries and biological functions. These include exemplary phosphomonoesters (NMPs, sugar phosphates, phosphoproteins, and inositol phosphates), phosphodiesters (DNA, RNA, phospholipids, and cyclic nucleotides), and phosphoanhydrides (ATP, NAD^+^, CoA, nucleoside diphosphate sugars, and inorganic pyrophosphate/polyphosphate). Phosphate availability is a key concern in ecology insofar as too much phosphate can promote unwanted cell proliferation (e.g., algal blooms), and too little phosphate can restrict growth (affecting crop yields). Cells from all domains of life respond to acute phosphate starvation by inducing the transcription of phosphate acquisition genes encoding secreted or cell surface-associated enzymes that mobilize phosphate from the extracellular environment, and transmembrane transporters of inorganic phosphate or simple phosphate-containing compounds. Different taxa rely on diverse strategies to achieve a rapid transcriptional response to acute phosphate starvation, on the time scale of a few hours.

Using the fission yeast *Schizosaccharomyces pombe* as a model eukaryote, we have begun to characterize the cellular responses to longer-term phosphate starvation in a time-resolved fashion, initially entailing integration of genome-wide and proteome-wide analyses of changes in mRNA and protein levels (1). *S. pombe* reacts to acute phosphate starvation (4 h) by upregulating genes with roles in phosphate acquisition and metabolism, autophagy genes, and a battery of core environmental stress response genes induced during many other forms of cellular stress (1, 2). There is an ~8 h lag between the acute transcriptional response and the time it takes for phosphate starvation to arrest growth, alter cell morphology, and establish a durable state of G0 quiescence (1). The transition to quiescence is temporally correlated with global downregulation of (i) genes responsible for synthesis and processing of rRNAs and tRNAs and ribosome assembly, (ii) nearly all genes encoding 60S and 40S ribosomal proteins, and (iii) genes encoding translation initiation factors. Proteome-wide liquid chromatography–tandem mass spectrometry (LC/MS-MS) analysis highlighted a depletion of the cellular pool of 60S and 40S ribosomal proteins during the transition to phosphate starvation-induced quiescence, consistent with the transcriptional downregulation of ribosomal protein gene expression. Concomitant with this ribosomal protein deficit, the 28S and 18S rRNAs became vulnerable to site-specific cleavages that generated temporally stable rRNA fragments. Despite these major alterations in physiology, fission yeast cells starved of phosphate for 2 days recover rapidly and resume growth when phosphate is replenished (1).

In the present study, we focus on how phosphate starvation impacts the fission yeast metabolome. The Yanagida lab has pioneered the metabolic profiling of fission yeast via LC/MS methods, especially in the context of nutrient limitations for glucose and nitrogen (3–5). However, little is known about the metabolic adaptations of fission yeast to phosphate starvation, beyond the fact that it results in rapid depletion (within 2 h) of the storage pool of vacuolar inorganic polyphosphate (1) and of the intracellular pools of inositol pyrophosphates 5-IP_7_, 1-IP_7_, and 1,5-IP_8_ (6). Here, we conducted a time-resolved metabolomics experiment in which we sampled fission yeast cells after 4, 8, 12, and 24 h of phosphate starvation versus cells grown in phosphate-replete medium. After establishing the scope and temporal order of starvation-induced metabolite changes, we proceeded to track the kinetics of metabolic recovery after cells starved for 24 h were replenished with phosphate-containing medium for 2, 4, 6, 8, and 12 h. Our findings provide a framework for understanding how phosphate status coordinately affects cell cycle, gene expression, and metabolism.

## RESULTS

### Metabolome profiling during phosphate starvation and phosphate replenishment

To profile the metabolome during phosphate starvation, fission yeast cells growing logarithmically at 30°C in Yeast Extract with Supplement (YES) medium were harvested, washed with water, and transferred to a customized rich synthetic medium ePMGT(–PO_4_) containing no phosphate, and then incubated at 30°C for 4, 8, 12, or 24 h. We had shown previously that upon transfer from YES to ePMGT(–PO_4_), fission yeast cells rapidly accumulate cell surface acid phosphatase (Pho1) and undergo two to three rounds of cell division before entering a state of G0 quiescence (1). RNA-seq of poly(A)^+^ RNA isolated from fission yeast cells prior to (time 0) and 4, 8, 12, and 24 h after transfer to ePMGT(–PO_4_) had identified 956 mRNAs that were upregulated (greater than or equal to a twofold increase in transcript read level) at two or more timepoints during the 24 h starvation interval and 926 mRNAs that were downregulated (greater than or equal to a twofold decrease in transcript read level) at two or more timepoints (see Table S1 for gene lists and log2 fold changes as a function of starvation time).

Phosphate-replete control cells were grown logarithmically for 24 h in ePMGT(+PO_4_) containing 15.5 mM phosphate. Fission yeast cells continue to grow normally after transfer from YES to ePMGT+PO_4_, i.e., the doubling time of 147 min in ePMGT(+PO_4_) is virtually identical to the doubling time of 143 min in YES, and there is no increase in cell surface acid phosphatase activity, a sensitive indicator of the phosphate starvation response (1). RNA-seq of poly(A)^+^ RNA isolated prior to (time 0) and 2, 4, and 8 h after transfer from YES to ePMGT(+PO_4_) flagged only 5 mRNAs that were downregulated by greater than or equal to twofold after transfer to ePMGT and 72 mRNAs that were upregulated (7). Forty-seven of the 72 genes up in ePMGT(+PO_4_) were also upregulated after transfer from YES to ePMGT(–PO_4_) (7). The fact that transfer from YES to ePMGT(+PO_4_) did not result in downregulation of the several hundred ribosome biogenesis, tRNA biogenesis, and protein translation genes seen during an equivalent interval after transfer from YES to ePMGT(–PO_4_) (1, 7) underscores that physiological differences between cells transferred to ePMGT(–PO_4_) versus ePMGT(+PO_4_) can be reasonably attributed to phosphate starvation *per se*.

To assess the metabolome during recovery from phosphate starvation, cells grown for 24 h in ePMGT(–PO_4_) medium were harvested, washed with ePMGT(+PO_4_), resuspended in ePMGT(+PO_4_), and incubated at 30°C for 2, 4, 6, 8, or 12 h. Cells grown for 24 h in ePMGT(+PO_4_) served as a phosphate-replete control. Six biological replicates of equivalent aliquots of cells (based on *A*_600_) were harvested for each condition by rapid filtration and immediate transfer of the membrane to an acetonitrile:methanol:water quench solution. Five replicates of equivalent-volume aliquots of ePMGT(+PO_4_) and ePMGT(–PO_4_) media were processed in parallel as controls. Metabolomic analysis by LC-MS was performed as described under Materials and Methods. A total of 177 standard-verified metabolites were identified, of which 49 were eliminated from consideration because they were present at similar levels in cell extracts and medium-only controls. The latter category includes all 20 amino acids, adenine, and uracil that were present in the ePMGT medium. The list of 128 metabolites that were considered is provided in Table S2.

The relative abundances of individual metabolites are plotted in bar graph format in the figures—as a function of starvation time (+PO_4_ for 24 h; –PO_4_ for 4, 8, 12, and 24 h, from left to right in each graph) and as a function of replenishment time after starvation (–PO_4_ for 24 h, then +PO_4_ for 2, 4, 6, 8, and 12 h; +PO_4_ for 24 h, from left to right in each graph), flanked on either side by the medium-only “blank” values. The replicate peak area values for the metabolites plotted in the figures are compiled in Table S3. We present below the findings as they pertain to certain metabolic pathways and/or classes of metabolites.

### Ribonucleotides

**Fig 1:**
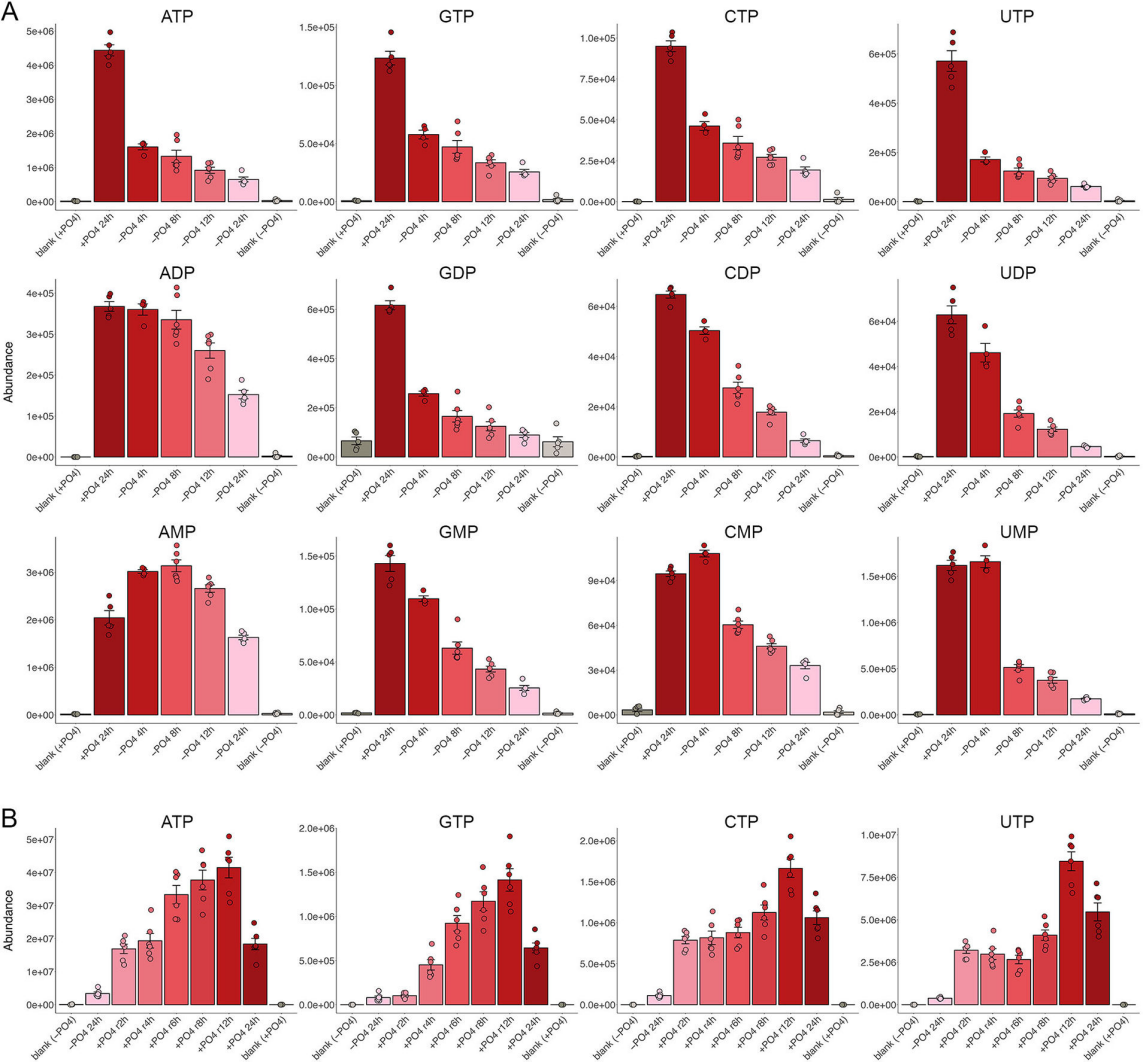
Changes in ribonucleotides during phosphate starvation and recovery. (**A**) The bar graphs show the levels of the indicated NTPs, NDPs, and NMPs in cells grown in phosphate-replete medium (+PO4 24 h) and cells maintained in phosphate-free medium (–PO4) for 4, 8, 12, and 24 h, as indicated on the x-axis. The +PO4 and –PO4 medium-only blank values are indicated in the leftmost and rightmost bars. (**B**) The bar graphs show the levels of the indicated NTPs in cells starved for phosphate for 24 h (–PO4 24 h) and in 24 h starved cells that were replenished with phosphate and allowed to recover (+PO4 r) for 2, 4, 6, 8, and 12 h. The –PO4 and +PO4 medium-only blank values are indicated in the leftmost and rightmost bars. Each bar height is the mean of the individual peak areas (circle symbols) ± SD. The shading of the bars, from dark-red to light-pink during phosphate starvation and from light-pink to dark-red during phosphate replenishment, is intended to make it easier for the viewer to follow the temporal progression of the metabolic changes during starvation and recovery.

The levels of ribonucleoside triphosphates ATP, GTP, CTP, and UTP were reduced by 60%–80% after 4 h of phosphate starvation and declined progressively thereafter, such that NTP levels after 24 h of starvation were 10%–20% of the phosphate-replete control values (Fig. 1A). Ribonucleoside diphosphates GDP, CDP, and UDP were reduced to a similar extent as the corresponding NTPs (to 14%, 10%, and 7% of the pre-starvation values after 24 h) and with roughly similar kinetics. By contrast, ADP levels were unaffected for the first 8 h of phosphate starvation and declined by only 60% after 24 h of starvation (Fig. 1A). Ribonucleoside monophosphates GMP, CMP, and UMP declined with slower kinetics vis-à-vis their cognate NDPs and NTPs. However, AMP levels increased during the first 12 h of phosphate starvation before returning to pre-starvation levels at 24 h. Thus, the ATP/AMP ratio was depressed throughout the starvation period. Calculation of the energy charge (EC) ratio, defined as ([ATP] +0.5[ADP])/([ATP] + [ADP] + [AMP]), based on the average peak areas for these metabolites yielded an EC value of 0.67 for control cells grown for 24 h in phosphate-replete ePMGT medium and EC values of 0.30 and 0.31 for cells subjected to 8 h and 24 h of phosphate starvation, respectively.

Restoration of phosphate to 24 h starved cells elicited a rapid rebound in ATP, CTP, and UTP levels by 2 h, approaching those in non-starved controls (Fig. 1B). GTP levels did not begin to increase until 4 h of phosphate replenishment. ATP and GTP increased progressively thereafter, attaining a level at 12 h that was higher than that of phosphate-replete cells that had not been starved and replenished (Fig. 1B). CTP and UTP levels at 12 h of recovery also exceeded those in non-starved controls.

### Deoxyribonucleoside triphosphates

**Fig 2:**
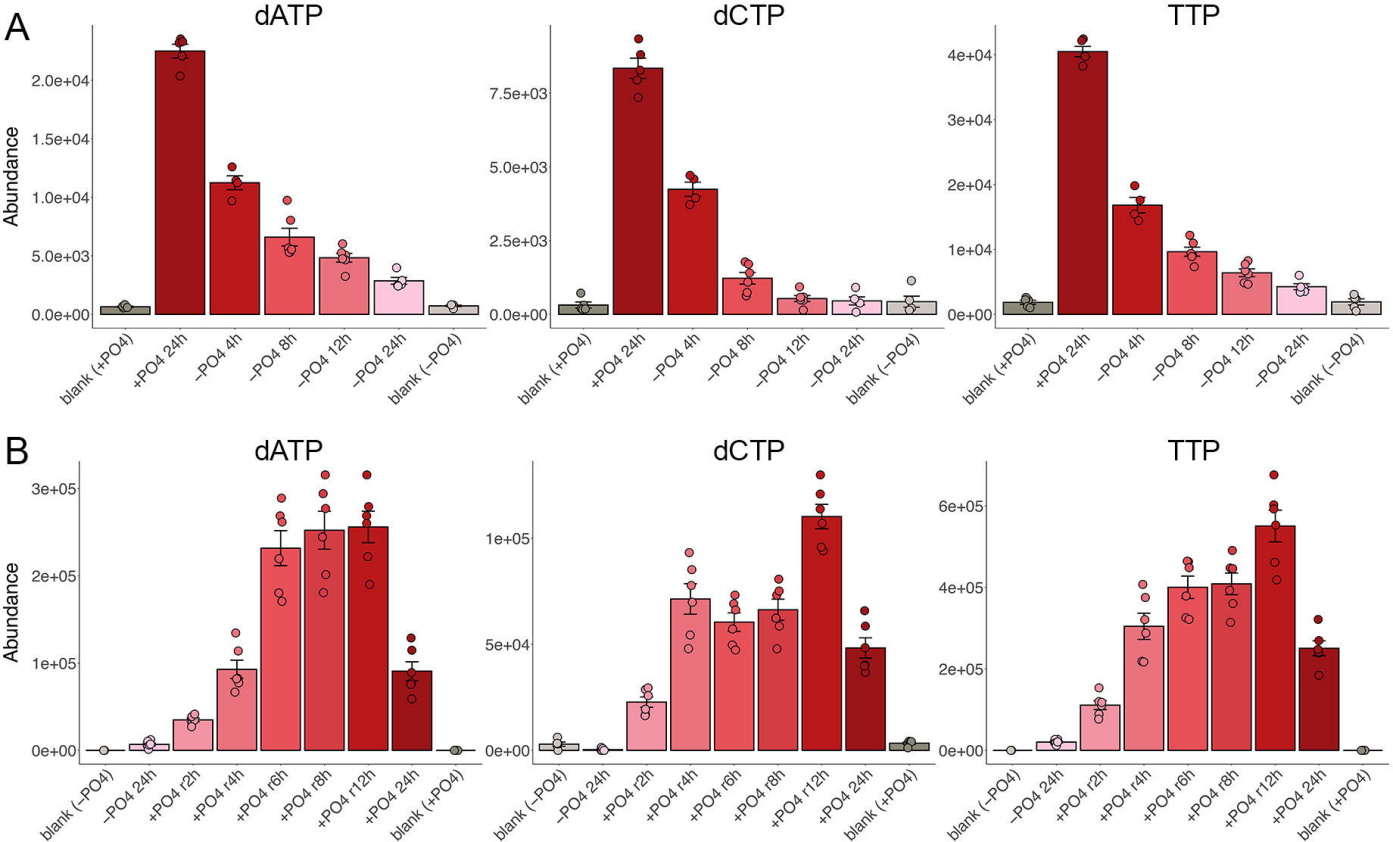
Changes in dNTPs during phosphate starvation and recovery. The data for dNTP levels during phosphate starvation (panel A) and recovery from phosphate starvation (panel B) are presented in bar graph format as described in the legend to Fig. 1.

dATP, dCTP, and TTP levels fell by half after 4 h of phosphate starvation and by an order of magnitude at 24 h (Fig. 2A). (We do not report on dGTP because it has the same molecular formula as the much more abundant ATP and cannot be distinguished readily by the methods used in this study.) The kinetics of dNTP depletion accord with the cessation of DNA replication and G0 cell cycle arrest after 24 h of phosphate starvation (1). dATP, dCTP, and TTP were restored to pre-starvation levels by 4 h after phosphate was replenished and exceeded pre-starvation levels after 12 h of recovery (Fig. 2B). The timing of dNTP pool repletion corresponds to the resumption of DNA replication and re-entry into the cell cycle that occurs at 6 to 8 h after starved cells (which are arrested in G0) are furnished with phosphate (1).

### *De novo* pyrimidine synthesis

**Fig 3:**
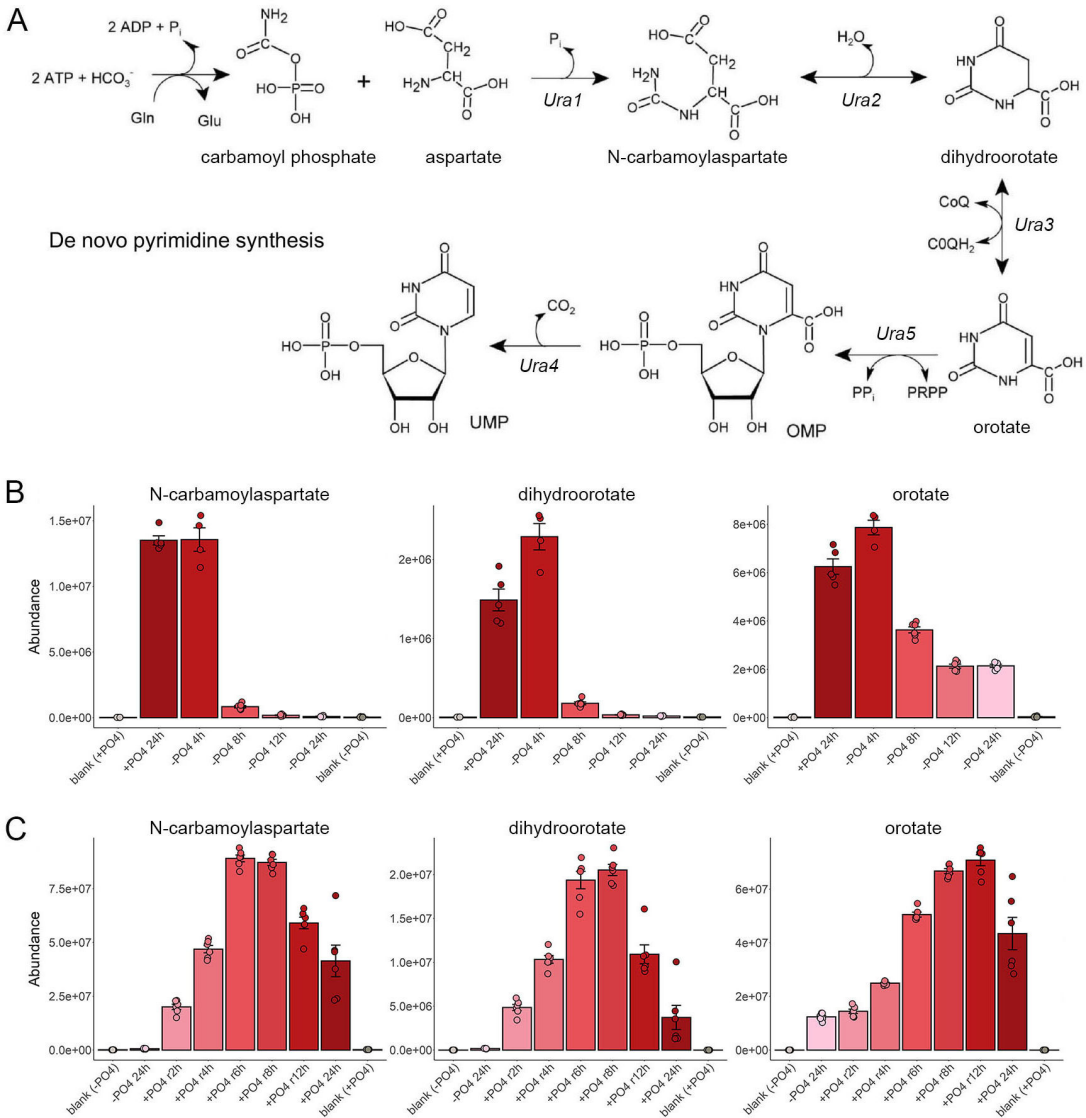
*De novo* pyrimidine synthesis during phosphate starvation and recovery. (**A**) The scheme of *de novo* pyrimidine nucleotide synthesis is shown. (**B, C**) The bar graphs depict the levels of serial pathway intermediates carbamoylaspartate, dihydroorotate, and orotate during phosphate starvation (panel B) and recovery from phosphate starvation (panel C).

The scheme of *de novo* pyrimidine nucleotide synthesis is shown in Fig. 3A. Our metabolomics experiments monitored the levels of serial pathway intermediates carbamoylaspartate, dihydroorotate, and orotate during phosphate starvation and recovery (Fig. 3B and C). At 4 h of starvation, upstream metabolites were either unaffected (carbamoylaspartate) or increased (dihydroorotate, orotate) (Fig. 3B) and the downstream end-product UMP was unaffected (Fig. 1A). Carbamoylaspartate and dihydroorotate levels declined acutely between 4 and 8 h of starvation, and the pools of these two metabolites were effaced at 12 h and 24 h of starvation, whereas orotate persisted at one-third of the pre-starvation level (Fig. 3B). The pools of dihydroorotate and carbamoylaspartate were restored to pre-starvation levels after 2 h and 4 h of phosphate replenishment, respectively, and all three pathway intermediates accumulated to levels that exceeded the pre-starvation state at 6 to 12 h of phosphate provision (Fig. 3C). Transcriptome profiling of phosphate-starved fission yeast indicated that the gene encoding dihydroorotate dehydrogenase Ura3 was downregulated by threefold (Table S1), whereas the mRNAs encoding the other four enzymes of *de novo* pyrimidine biosynthesis were unaffected (1).

### Glycolysis

**Fig 4:**
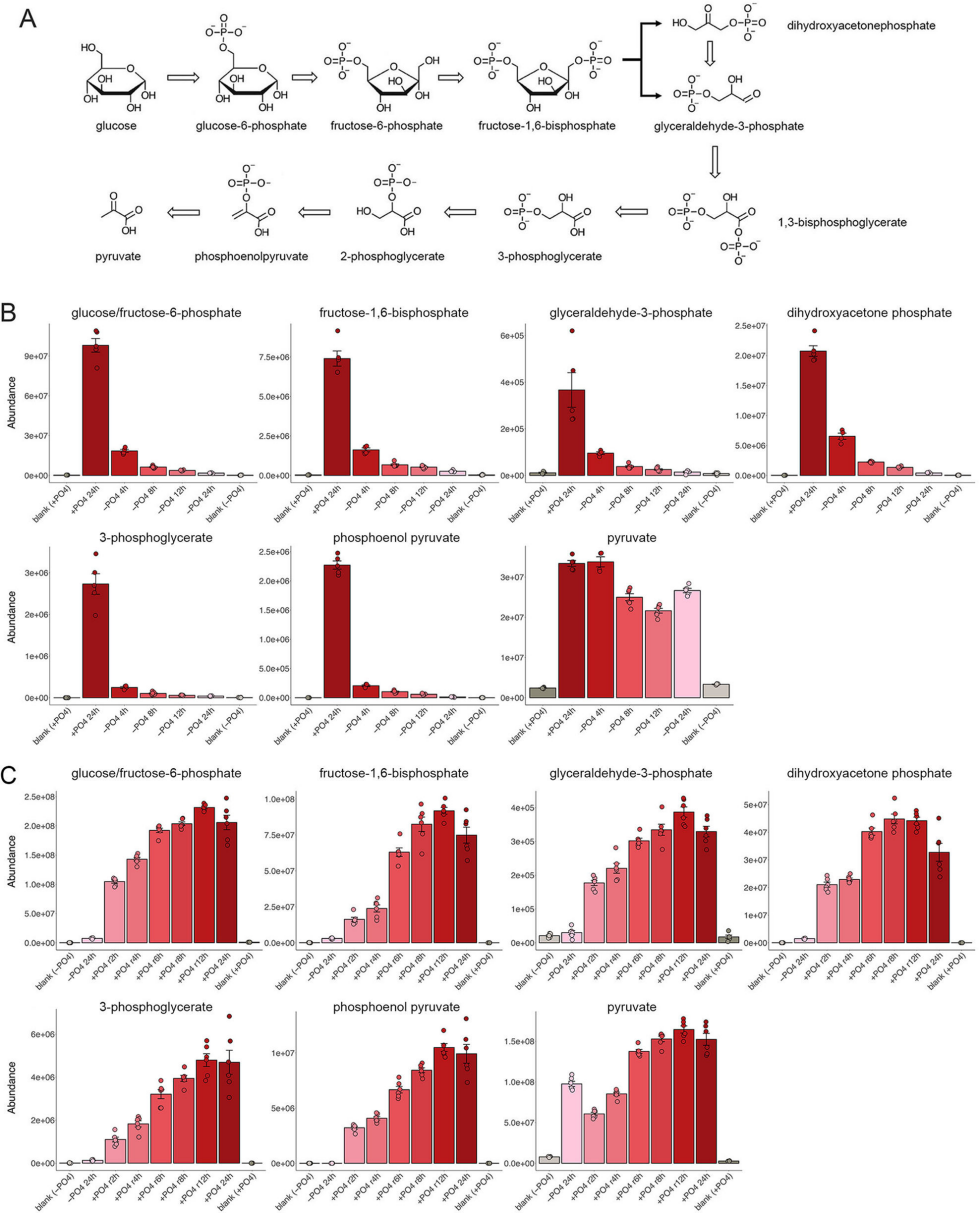
Glycolysis during phosphate starvation and recovery. (**A**) The glycolysis pathway is shown. (**B, C**) The bar graphs depict the levels of serial glycolysis pathway intermediates glucose/fructose-6-phosphate, fructose-1,6-bisphosphate, glyceraldehyde-3-phosphate, 3-phosphoglycerate, and phosphoenolpyruvate and the pathway end-product pyruvate during phosphate starvation (panel B) and recovery from phosphate starvation (panel C).

Phosphate starvation profoundly affected the pathway of glycolysis (Fig. 4A). To wit, pathway intermediates glucose/fructose-6-phosphate, fructose-1,6-bisphosphate, glyceraldehyde-3-phosphate, 3-phosphoglycerate, and phosphoenolpyruvate all regressed deeply at 4 h of starvation and were virtually erased at 24 h (Fig. 4B). Each of these glycolytic metabolites was regenerated to pre-starvation levels during 6 to 12 h of phosphate replenishment (Fig. 4C). Our analysis of the fission yeast transcriptome during 24 h of phosphate starvation revealed significant changes in mRNAs encoding glycolytic enzymes, whereby (i) hexokinase Hxk2 was upregulated by 5-fold; (ii) glucose-6-phosphate isomerase Pgi1 was upregulated by 2-fold; (iii) phosphofructokinase Pfk1 was upregulated by 3-fold; (iv) fructose-bisphosphate aldolase Fba1 was upregulated by 6-fold; (v) triosephosphate isomerase Tpi1 was upregulated by 4-fold; (vi) glyceraldehyde-3-phosphate dehydrogenase enzymes Gpd3 and Tdh1 were upregulated by 75-fold and 10-fold, respectively; (vii) phosphoglycerate kinase Pgk1 was upregulated by 8-fold; (viii) phosphoglycerate mutase Gpm1 was upregulated by 8-fold; (ix) enolases Eno102 and Eno101 were upregulated by 70-fold and 6-fold, respectively; and (x) pyruvate kinase Pyk1 was upregulated by 8-fold (1) (Table S1). The large fold increases in the *gpd3* and *eno102* mRNAs during phosphate starvation correlated with proteomics data revealing 12-fold and 16-fold increases in intracellular Gpd3 and Eno102, respectively (1). It was noteworthy that pyruvate, the end-product of glycolysis, was largely unaffected by phosphate starvation (Fig. 4B). It is conceivable that transcriptional upregulation of every glycolytic enzyme upstream of pyruvate during phosphate starvation enhances flux through the pathway, leading to preserved net pyruvate formation. Alternatively, pyruvate levels might be maintained via a non-glycolytic route in which late Krebs cycle intermediates malate or oxaloacetate are converted to pyruvate by malic enzyme(s). In this vein, it is remarkable that the malic enzyme Mae2 (oxaloacetate decarboxylating) is transcriptionally upregulated by 30-fold during phosphate starvation (1) (Table S1).

### Pentose phosphate pathway

**Fig 5:**
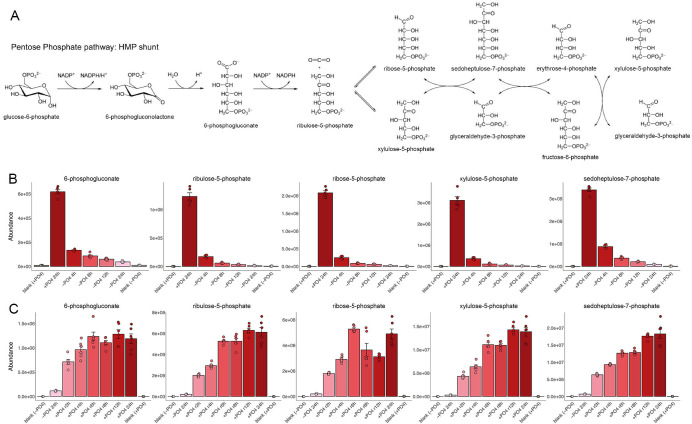
Pentose phosphate pathway. (**A**) The pentose phosphate pathway, also known as the hexose monophosphate (HMP) shunt, is shown. (**B, C**) The bar graphs depict the levels of serial pathway intermediates 6-phosphogluconate, ribulose-5-phosphate, ribose-5-phosphate, xylulose-5-phosphate, and sedoheptulose-7-phosphate during phosphate starvation (panel B) and recovery from phosphate starvation (panel C).

The pentose phosphate pathway (aka hexose monophosphate shunt) utilizes glucose-6-phosphate generated in the first step of glycolysis to ultimately form fructose-6-phosphate and glyceraldehyde-3-phosphate via a series of oxidative and non-oxidative steps (Fig. 5A). The pentose phosphate pathway provides two vital anabolic metabolites: NADPH required for fatty acid synthesis and ribose-5-phosphate required for nucleotide and nucleic acid synthesis. We found that serial pathway intermediates 6-phosphogluconate, ribulose-5-phosphate, ribose-5-phosphate, xylulose-5-phosphate, and sedoheptulose-7-phosphate were rapidly depleted in lockstep during phosphate starvation (Fig. 5B) and reconstituted during phosphate rescue (Fig. 5C). Transcription profiling revealed that the expression levels of genes encoding enzymes of the pentose phosphate pathway were affected as follows: (i) glucose-6-phosphate dehydrogenases Gcd1 and SPAC3C7.13c were upregulated by sixfold and fivefold, respectively; (ii) 6-phosphogluconate dehydrogenase Gnd1 was upregulated by threefold; (iii) ribose-5-phosphate isomerase Rki1 was downregulated by threefold; and (iv) transaldolase Tal1 was upregulated by threefold (1) (Table S1).

### Krebs cycle

**Fig 6:**
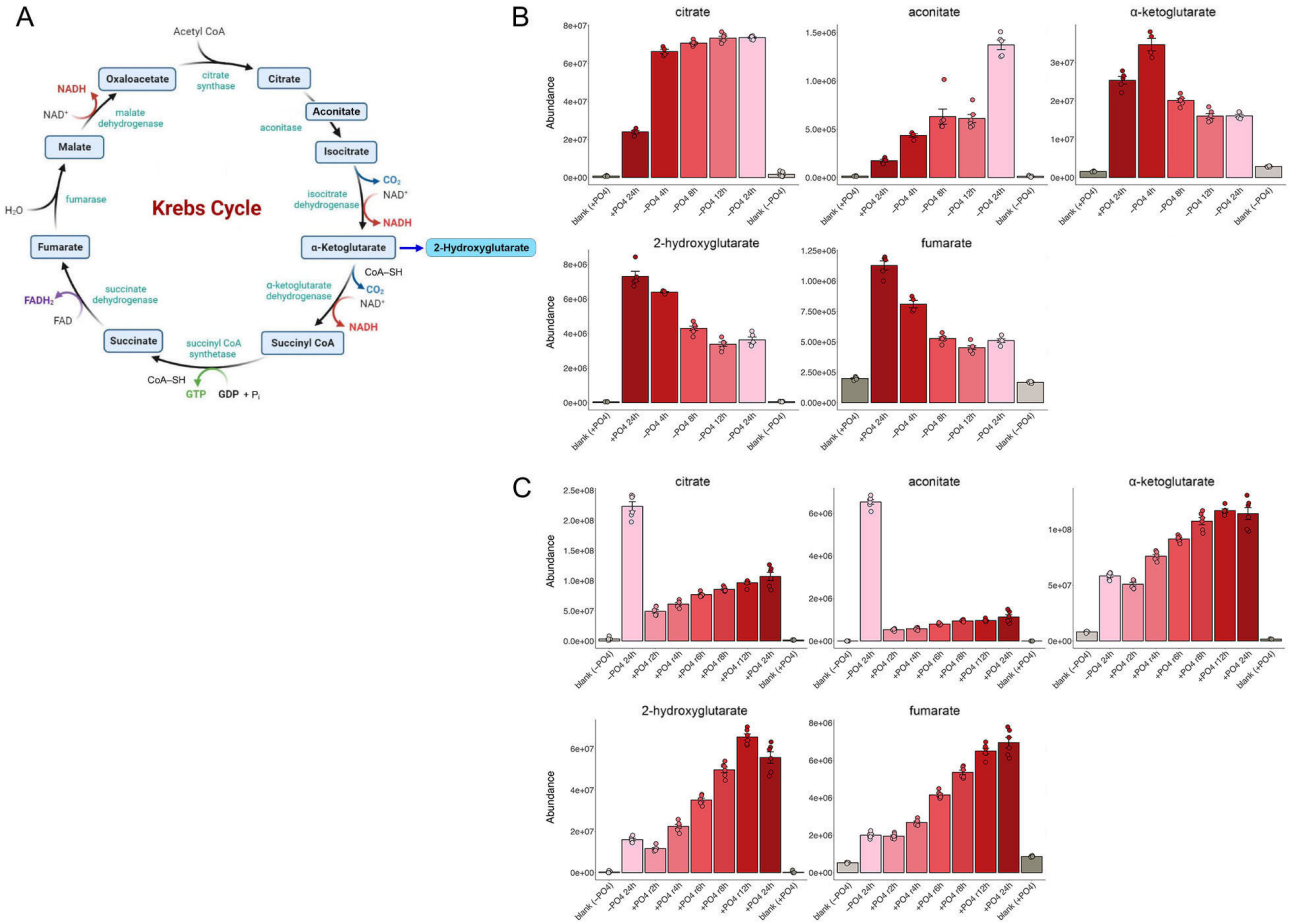
Krebs cycle. (**A**) The Krebs cycle is shown. The image is a modified version of that available online at https://microbenotes.com/krebs-cycle. (**B, C**) The bar graphs depict the levels of serial pathway intermediates citrate, aconitate, α-ketoglutarate, and fumarate—and by-product 2-hydroxyglutarate—during phosphate starvation (panel B) and recovery from phosphate starvation (panel C).

Phosphate starvation elicited a sustained increase in the levels of early Krebs cycle intermediates citrate/isocitrate and aconitate (Fig. 6A and B). By contrast, downstream Krebs cycle intermediates and derivatives α-ketoglutarate, 2-hydroxyglutarate, and fumarate were reduced modestly. (Oxaloacetate is extremely unstable and cannot be measured by standard metabolomics methods.) Transcription profiling showed that phosphate starvation affected the expression of genes encoding Krebs cycle enzymes as follows: (i) citrate synthase Cit1 was upregulated by fourfold; (ii) aconitases Aco1 and Aco2 were unaffected; (iii) isocitrate dehydrogenases Idh1 and Idh2 were upregulated by threefold; (iv) oxoglutarate dehydrogenase complex subunit Kgd2 was upregulated by threefold; and (v) succinate dehydrogenase subunit Sdh1 was upregulated by threefold (1) (Table S1). The high levels of citrate/isocitrate and aconitate generated after 24 h of starvation were reduced to less than pre-starvation levels after 2 h of phosphate replenishment and then steadily returned to the pre-starvation state (Fig. 6C). The reduction in α-ketoglutarate, 2-hydroxyglutarate, and fumarate seen after 24 h of starvation was rectified over 8 to 12 h of phosphate replenishment (Fig. 6C).

### NAD^+^ and derivatives

**Fig 7:**
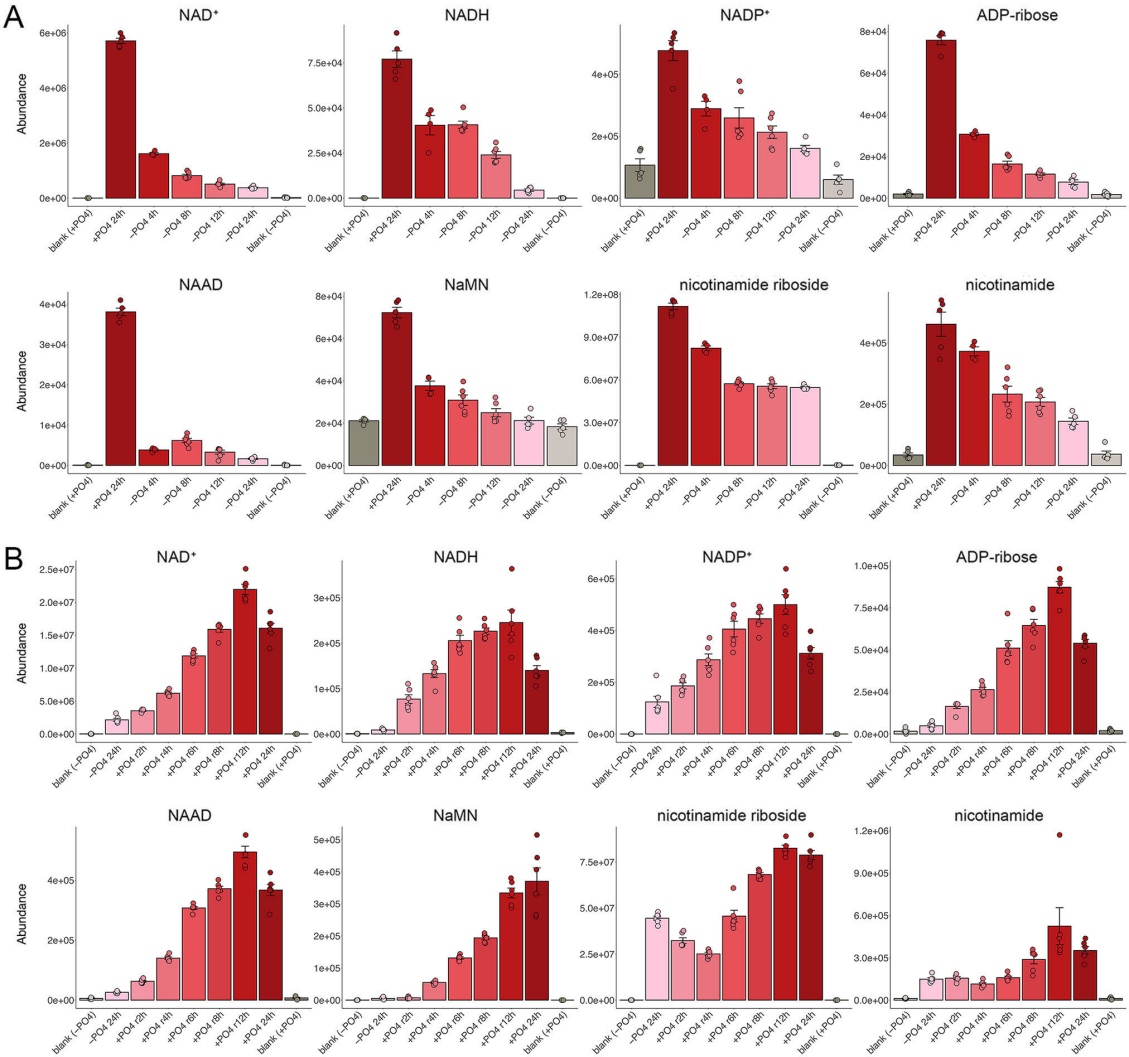
NAD^+^ and derivatives. The bar graphs depict the levels of NAD^+^, NADH, NADP^+^, ADP-ribose, NAAD, NaMN, nicotinamide riboside, and nicotinamide during phosphate starvation (panel A) and recovery from phosphate starvation (panel B).

NAD^+^ is synthesized from nicotinic acid mononucleotide (NaMN) formed either by multi-step *de novo* synthesis from tryptophan or via salvage. Nicotinate/nicotinamide mononucleotide adenylyltransferases (NMATs) catalyze the reaction of NaMN and ATP to form nicotinic acid adenine dinucleotide (NAAD) and pyrophosphate (PP_i_). NAAD is then converted by glutamine-dependent synthase Qns1 to NAD^+^. The intersecting salvage pathway from nicotinamide entails conversion of nicotinamide to nicotinic acid by Pnc1 and ensuing Npt1-catalyzed reaction of nicotinic acid with PRPP to form NaMN. Alternatively, NAD^+^ is generated via salvage from nicotinamide riboside, whereby kinase Nrk1 converts nicotinamide riboside to nicotinamide mononucleotide, which NMAT converts to NAD^+^. Here, we observed that intracellular NAD^+^ was reduced by 72% after 4 h of phosphate starvation and continued to decline thereafter, to 7% of pre-starvation levels after 24 h (Fig. 7A). Its immediate precursors NAAD and NaMN displayed similar patterns of depletion (Fig. 7A). Salvage pathway substrates nicotinamide and nicotinamide riboside declined modestly, by threefold and twofold, respectively, during the 24 h starvation period (Fig. 7A). Transcriptome profiling indicated that expression of the genes encoding the aforementioned NAD^+^-forming enzymes was unaffected by phosphate starvation, except for the SPAC694.03 gene encoding NMAT, which was upregulated by fourfold (1) (Table S1). Downstream NAD^+^ metabolites NADH, NADP^+^, and ADP-ribose declined over time in near lockstep with NAD^+^ (Fig. 7A). Of the four fission yeast genes encoding an NAD/NADH kinase enzyme, only SPCC24B10.02 expression was altered (increased by threefold) during phosphate starvation (1) (Table S1). The cellular pool of NAD^+^, its precursors NAAD and NaMN, and its metabolites NADH, NADP^+^, and ADP-ribose were restored progressively during the 12 h period of phosphate replenishment (Fig. 7B).

### Nucleotide sugars

**Fig 8:**
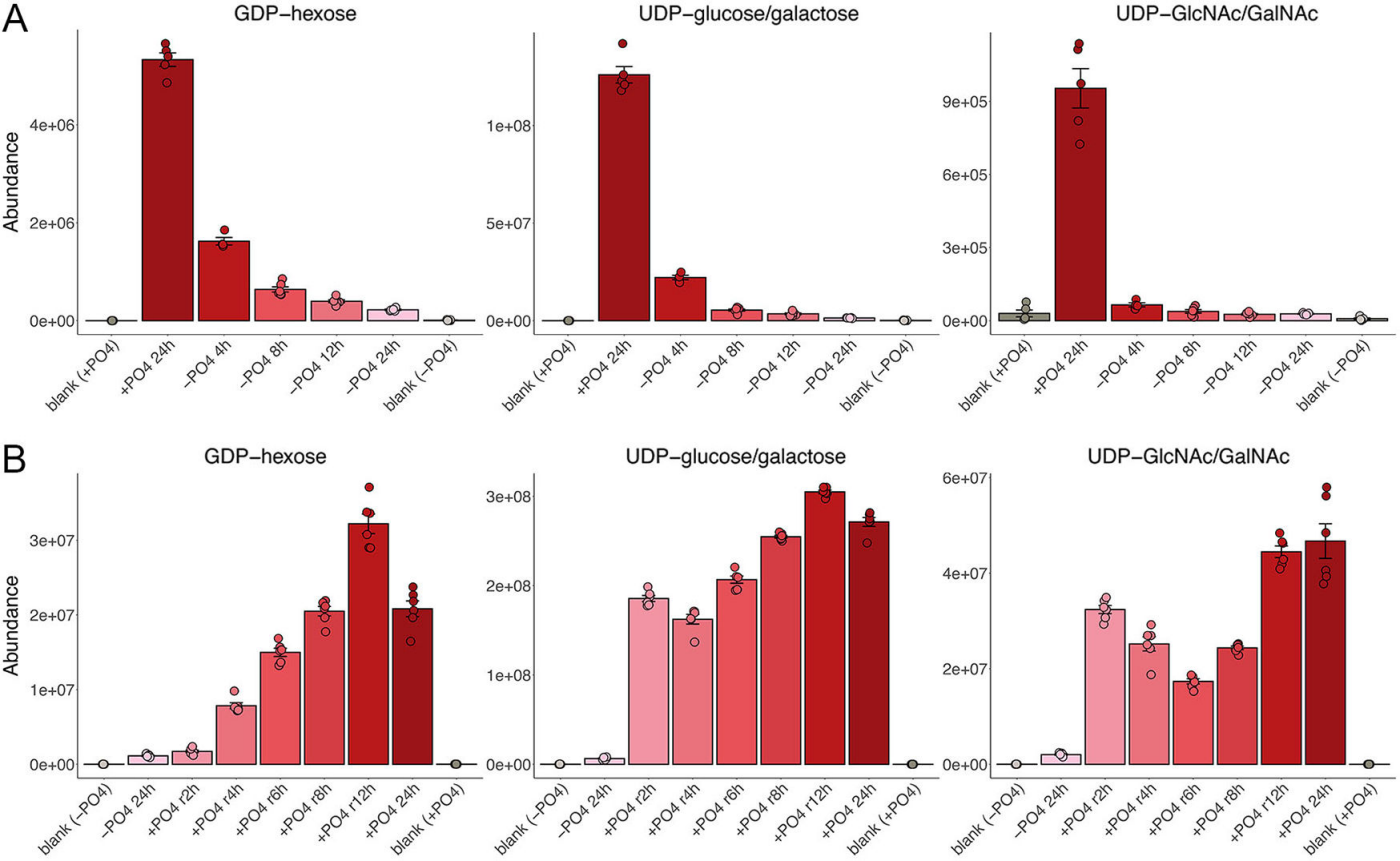
Nucleotide sugars. The bar graphs depict the levels of GDP-hexose, UDP-glucose/galactose, and UDP-GalNAc/GlcNAc during phosphate starvation (panel A) and recovery from phosphate starvation (panel B).

Nucleoside diphosphate sugars are the activated forms of monosaccharides that function as donors in glycosylation reactions. They are formed by condensation of an NTP with a monosaccharide-1-phosphate to form an NDP-sugar and PP_i_. We found that phosphate starvation resulted in a rapid decrement in nucleotide sugars GDP-hexose, UDP-glucose/galactose, and UDP-GalNAc/GlcNAc (Fig. 8A). These nucleotide sugar pools were reconstituted during recovery from phosphate starvation (Fig. 8B). At the transcriptional level, the expression of Fyu1, an enzyme that synthesizes UDP-glucose, is upregulated by sevenfold during phosphate starvation (1) (Table S1).

### AdoMet and derivatives and polyamine synthesis

**Fig 9:**
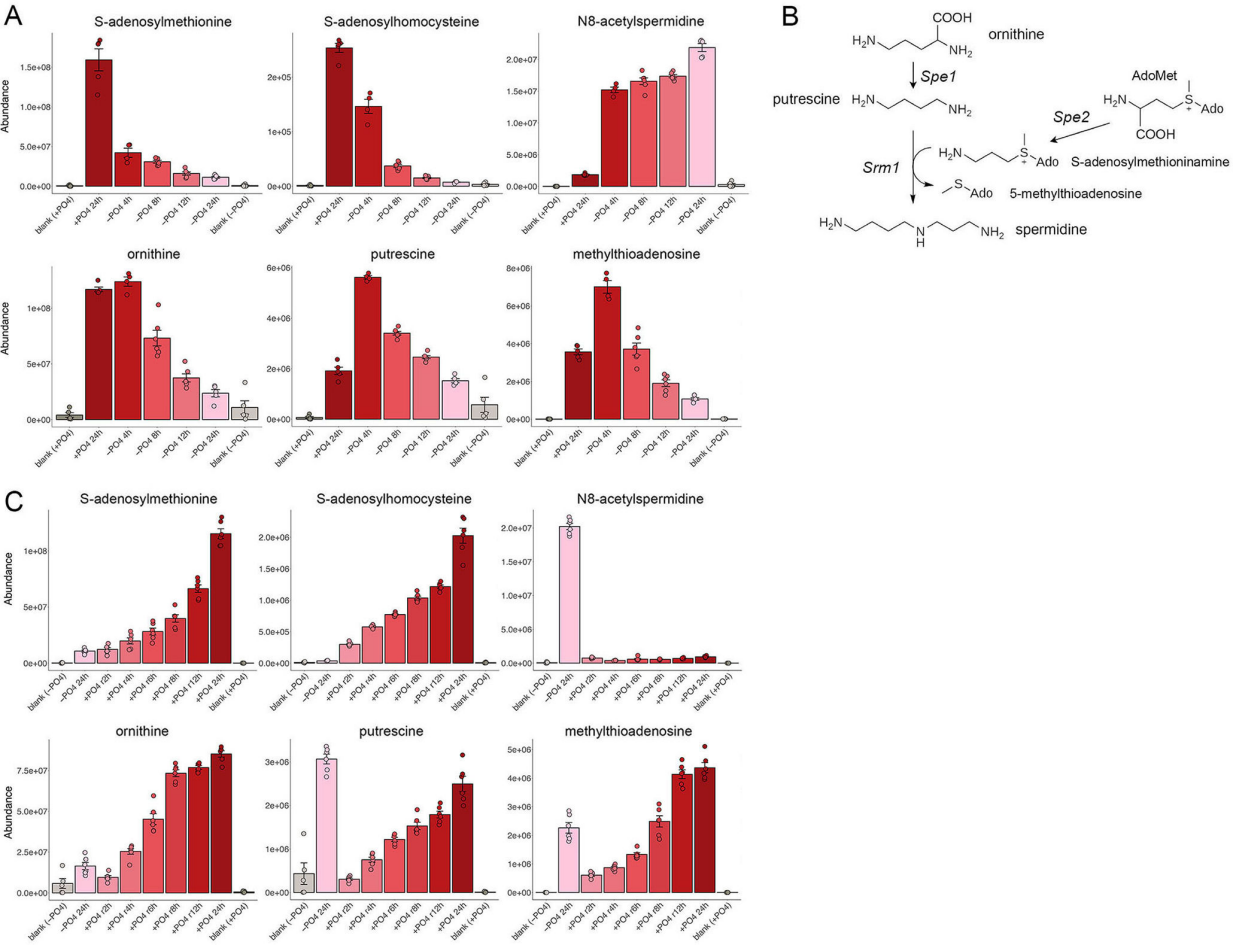
AdoMet and derivatives and polyamine synthesis. The pathway of spermidine synthesis from ornithine is depicted in panel B. The bar graphs show the levels of *S*-adenosylmethionine, *S*-adenosylhomocysteine, ornithine, putrescine, methylthioadenosine, and N8-acetylspermidine during phosphate starvation (panel A) and recovery from phosphate starvation (panel C).

*S*-Adenosylmethionine (AdoMet) is a donor for methyl group transfer to a multitude of methyl acceptors (nucleic acids, proteins, lipids, and metabolites). *S*-Adenosylhomocysteine (AdoHcy) is the product of AdoMet-dependent transmethylation. Phosphate starvation resulted in progressive depletion of both AdoMet and AdoHcy (Fig. 9A). AdoMet synthesis by methionine adenosyltransferase Sam1 entails reaction of ATP and methionine to form AdoMet, PP_i_, and P_i_. Given that methionine is present at ample levels in ePMGT medium, it is likely that the decline in AdoMet reflects the decline in ATP during phosphate starvation (Fig. 1A), abetted by a fourfold decrease in the mRNA encoding Sam1 (1) (Table S1). AdoMet is also the source of a key substrate for synthesis of the polyamine spermidine (Fig. 9B). Polyamine synthesis initiates with the decarboxylation of ornithine to putrescine (by Spe1) and the decarboxylation of AdoMet to *S*-adenosylmethioninamine (by Spe2). Spermidine synthase Srm1 then catalyzes the reaction of putrescine with *S*-adenosylmethioninamine to form 5-methylthioadenosine and spermidine (Fig. 9B). Upstream precursor ornithine was unaffected at 4 h of phosphate starvation and declined steadily thereafter (Fig. 9A). By contrast, intermediates putrescine and 5-methylthioadenosine increased at 4 h before declining at later times (Fig. 9A). Although our experiment did not directly measure spermidine or spermine, a noteworthy finding was that the derivative N8-acetylspermidine increased markedly by 4 h of starvation and remained elevated for 24 h (Fig. 9A). At the transcriptional level, the mRNAs encoding Spe2 and Srm1 are both downregulated by fourfold during phosphate starvation (1) (Table S1). AdoMet, AdoHcy, and ornithine levels recovered gradually over time after cells starved for 24 h were replenished with phosphate (Fig. 9C). Putrescine and 5-methylthioadenosine declined sharply after 2 h of phosphate replenishment before gradually recovering (Fig. 9C). The N8-acetylspermidine that accumulated during starvation was rapidly depleted within 2 h of phosphate replenishment (Fig. 9C).

### Vitamins and derivatives

**Fig 10:**
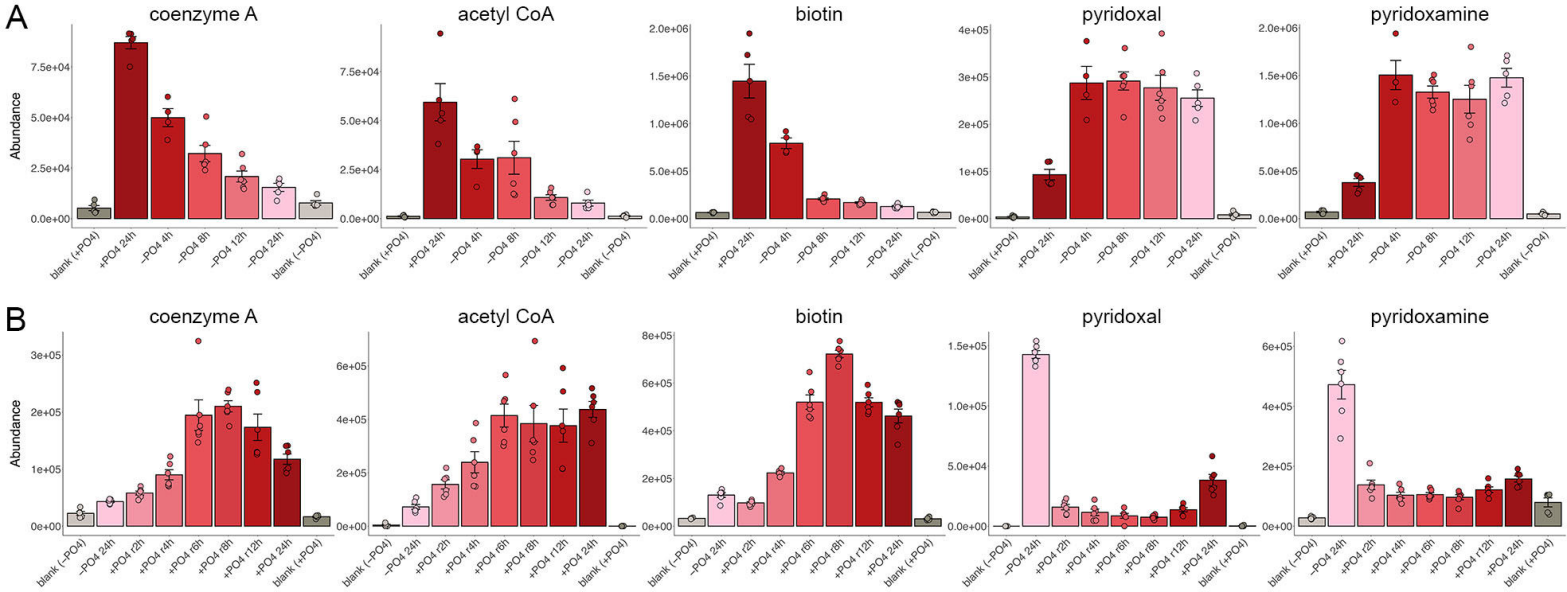
Vitamins and derivatives. The bar graphs show the levels of coenzyme A, acetyl CoA, biotin, pyridoxal, and pyridoxamine during phosphate starvation (panel A) and recovery from phosphate starvation (panel B).

Coenzyme A (CoA), acetyl CoA, and biotin levels decreased steadily with the duration of phosphate starvation (Fig. 10A) and were restored over time during phosphate replenishment (Fig. 10B). By contrast, pyridoxal and pyridoxamine increased after 4 h of starvation and remained elevated for 24 h (Fig. 10A) and then declined to pre-starvation levels (or less) after 2 h of phosphate replenishment (Fig. 10B).

### Choline metabolism

**Fig 11:**
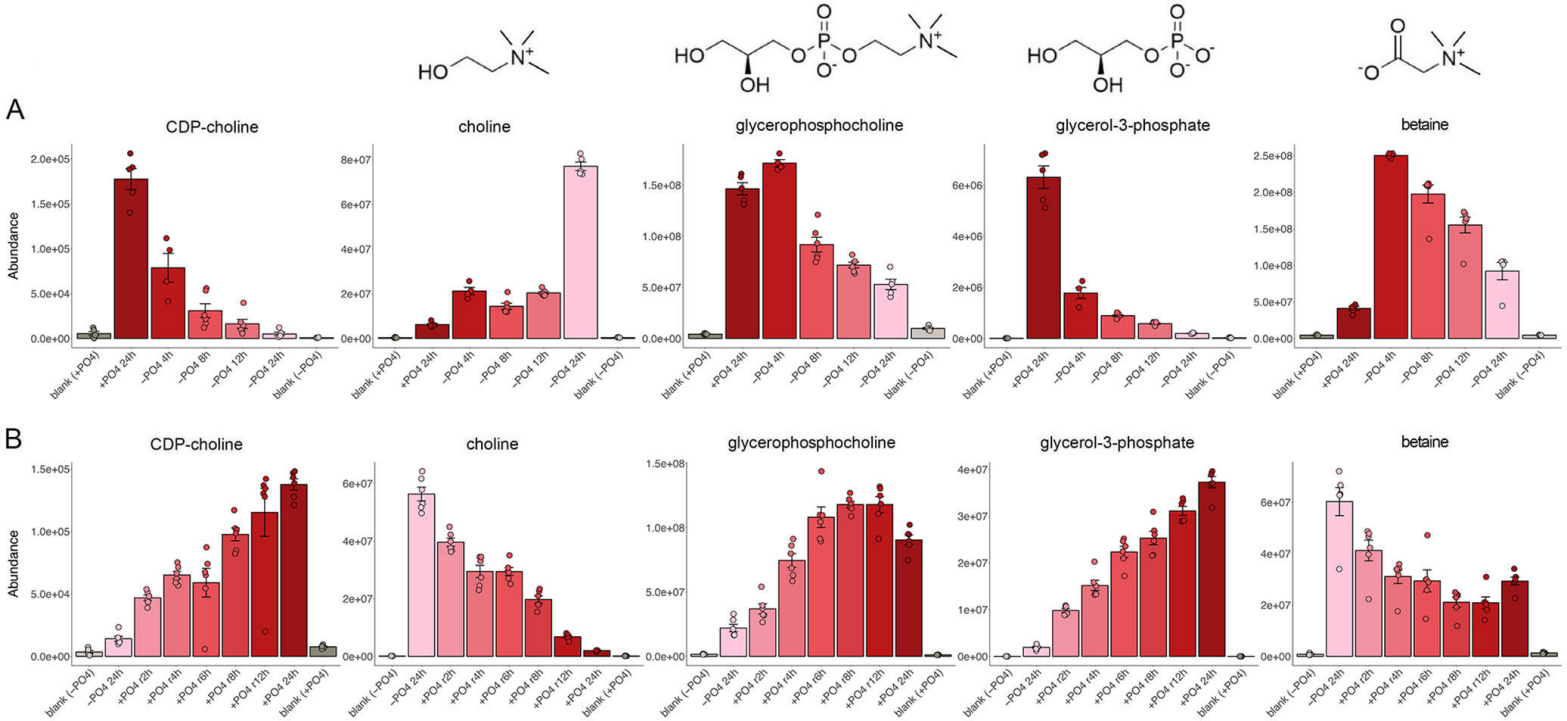
Choline metabolism. The bar graphs depict the levels of CDP-choline, choline, glycerophosphocholine, glycerol-3-phosphate, and betaine during phosphate starvation (panel A) and recovery from phosphate starvation (panel B). Structures of the metabolites are shown above the graphs.

CDP-choline, a key intermediate in the Kennedy pathway of glycerophosphocholine lipid synthesis, declined progressively during phosphate starvation and was virtually eliminated at 24 h (Fig. 11A). CDP-choline is formed in two steps: (i) choline kinase Eki1 converts choline to phosphocholine and (ii) Pcy1 transfers CMP from CTP to phosphocholine to generate CDP-choline. The mRNA encoding choline kinase Eki1 is downregulated by sixfold during phosphate starvation, whereas the mRNA encoding cytidyltransferase Pcy1 is upregulated by twofold (1) (Table S1). An alternative pathway of glycerophosphocholine lipid synthesis entails two sequential acylations of glycerophosphocholine (GPC) (8). GPC is generated intracellularly by deacylation of phosphatidylcholine catalyzed by phospholipase B enzymes. Phosphate starvation resulted in a transient increase in GPC at 4 h followed by a progressive decline to one-third of the pre-starvation level at 24 h (Fig. 11A). The mRNA encoding phospholipase B Plb1 is increased by threefold after 4 h of starvation (1) (Table S1). GPC is hydrolyzed to choline and glycerol-3-phosphate by glycerophosphodiesterase Gde1. The mRNA encoding Gde1 increases by twofold during starvation (Table S1). Whereas choline levels increased by 2- to 3-fold at 4–12 h of phosphate starvation and then by 12-fold at 24 h, glycerol-3-phosphate fell by 72% at 4 h and by 97% at 24 h (Fig. 11A). Glycerol-3-phosphate can be oxidized to dihydroxyacetone phosphate by glycerol-3-phosphate dehydrogenase Gpd1, which is transcriptionally upregulated by eightfold during phosphate starvation (1) (Table S1). A striking finding was that betaine (trimethylglycine), a metabolite derived from choline via a betaine dialdehyde intermediate, was increased by sixfold at 4 h of phosphate starvation and remained elevated for 24 h vis-à-vis pre-starvation level (Fig. 11A). We attribute the accumulation of betaine to the phosphate starvation-induced 100-fold increase in the mRNA encoding aldehyde dehydrogenase Atd1 (1) (Table S1), an enzyme that converts betaine aldehyde to betaine.

### Lysine metabolism

**Fig 12:**
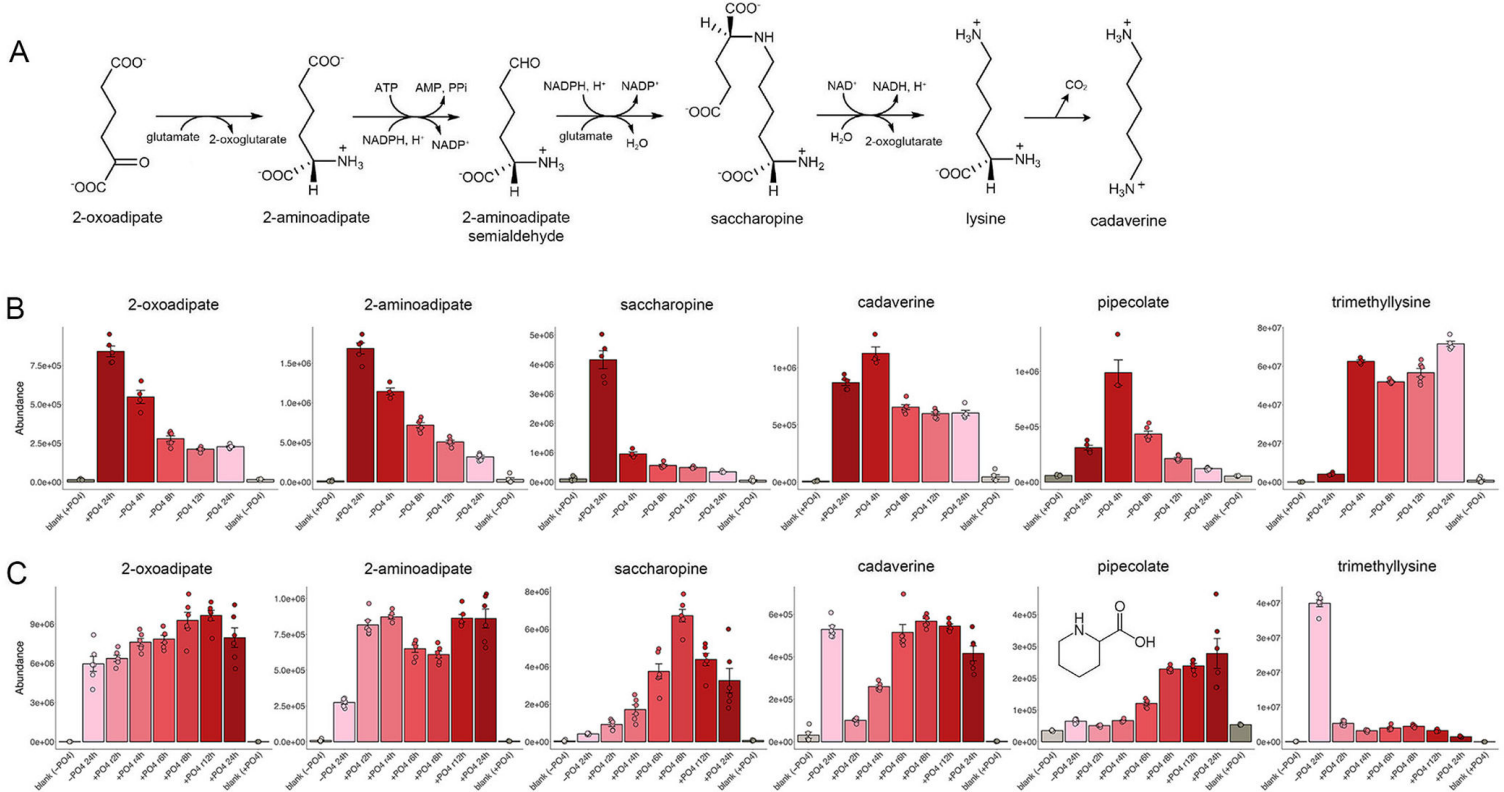
Lysine metabolism. (**A**) The final steps in the pathway of lysine synthesis from α-ketoglutarate via the aminoadipate pathway are shown, as is the decarboxylation of lysine to cadaverine. (**B, C**) The bar graphs depict the levels of serial aminoadipate pathway intermediates 2-oxoadipate, 2-aminoadipate, and saccharopine and by-products of lysine metabolism cadaverine, pipecolate, and trimethyllysine during phosphate starvation (panel B) and recovery from phosphate starvation (panel C).

Fission yeast synthesize lysine *de novo* from α-ketoglutarate via the aminoadipate pathway (9), the later steps of which are shown in Fig. 12A. Although ePMGT medium contains ample lysine, we found that phosphate starvation resulted in a time-dependent depletion of aminoadipate pathway intermediates 2-oxoadipate, 2-aminoadipate, and saccharopine (Fig. 12B). The level of α-ketoglutarate (the start of the pathway) is not grossly affected by starvation (Fig. 6B), and the mRNAs encoding the Lys4, Lys2, Lys12, and Lys1/Lys7 enzymes that generate saccharopine are not altered (1). The mRNA encoding Lys3, which converts saccharopine to lysine, is downregulated by twofold during phosphate starvation (1) (Table S1). It is possible that the observed declines in lysine synthetic intermediate reflect decrements in ATP, NAD^+^, and NADPH that serve as co-substrates for the enzymes in the pathway.

Phosphate starvation also resulted in transient changes in products of lysine catabolism, whereby cadaverine and pipecolate are increased after 4 h of starvation before declining at later times (Fig. 12B). Note that the mRNA encoding L-pipecolate oxidase Fap1, an enzyme that generates pipecolate from ∆^1^-piperideine-6-carboxylate, is upregulated by threefold during phosphate starvation (1) (Table S1).

Finally, phosphate starvation was accompanied by a rapid and sustained increase in trimethyllysine (Fig. 12B), which declined rapidly to pre-starvation levels upon phosphate replenishment (Fig. 12C). Trimethyllysine is a post-translational protein modification, catalyzed by AdoMet-dependent methyltransferases, that exerts epigenetic effects on gene expression. Free trimethyllysine amino acid (Kme3) is produced by proteolysis of Kme3-modified proteins (and potentially by methylation of free lysine) and is the initial substrate in a pathway of carnitine biosynthesis.

## DISCUSSION

Here, we characterized the metabolic adaptions of fission yeast to phosphate starvation and phosphate replenishment after starvation, in a time-resolved fashion. The 24 h phosphate starvation interval that we interrogated entails many “moving parts” that eventuate in G0 cell cycle arrest, induction of autophagy, and coherent changes in the transcriptome, especially the upregulation of genes involved in phosphate mobilization and the downregulation of genes that produce, assemble, and modify the rRNA, tRNA, ribosomal proteins, and translation initiation factors that comprise the cellular translation machinery (1). Despite the depth and breadth of these changes, fission yeast cells deprived of phosphate for 24 h suffer no loss of viability and resume DNA replication and cell division when restored to phosphate-containing medium, with virtually the same kinetics as they ceased dividing during phosphate starvation (1). To a first approximation, we find that the same held true for metabolome dynamics in phosphate-starved cells, i.e., that many of the time-dependent changes in individual metabolites and/or metabolic pathways elicited during phosphate starvation were reversed over a similar time frame during phosphate replenishment.

Given the centrality of phosphate to energetics and macromolecular synthesis, it was no surprise that a wide variety of phosphate-containing metabolites were progressively depleted during phosphate starvation. Prominent among these were the rNTP and dNTP precursors for transcription and DNA replication, respectively, and ATP, CoA, NAD^+^, NADP^+^, and NADH, which are co-substrates for many of the enzymes of intermediary metabolism.

Phosphate starvation affected multiple metabolic pathways, entailing either (i) pan-depletion of pathway intermediates (glycolysis, pentose phosphate pathway, and lysine synthesis), (ii) transient or sustained accumulation of early pathway intermediates (pyrimidine synthesis, Krebs cycle, and polyamine synthesis), or (iii) transient or sustained accumulation of catabolized or modified derivates of pathway products (betaine, choline, acetylspermidine, pipecolate, and trimethyllysine).

The causes and effects of these metabolome alterations are undoubtedly multifactorial and difficult to decipher. Among the likely determinants of changes in metabolite levels are depletion of the aforementioned phosphate-containing co-substrates for the enzymes that catalyze phosphoryl transferase and oxidoreductase reactions. It is to be expected that ATP-dependent or NAD(P)^+^-dependent enzymes with different affinities (*K*_m_) for their co-substrates might be differentially affected as the concentrations of their co-substrates decline over time during phosphate starvation. Changes in the levels of feedback inhibitors or allosteric regulators of enzyme activity during phosphate starvation can also impact metabolic pathways. A decline in the intracellular concentration of inorganic phosphate is likely, *per se*, to affect many enzymatic reactions for which phosphate is either a substrate or a product. As an example of the latter, it is thought that the rapid depletion of IP_8_ during phosphate starvation of fission yeast is attributable, at least in part, to mitigation of phosphate-dependent inhibition of pyrophosphatase enzymes that catabolize IP_8_ (6, 10–13). Moreover, the yeast kinase enzyme that synthesizes IP_8_ is subject to an acute loss of activity when the concentration of the ATP phosphodonor substrate declines (13), as is the case during phosphate starvation.

An additional layer of complexity is that the metabolic shifts documented here are occurring in parallel with an extensive remodeling of the fission yeast transcriptome (1). Indeed, as we pointed out in the Results section, phosphate starvation significantly increases or decreases the expression of mRNAs encoding many of the enzymes that form the metabolites that are affected by phosphate starvation. This raises a chicken-and-egg conundrum. Are the changes in expression of genes encoding metabolic enzymes triggered in response to fluctuations in the levels of their substrates, products, or other pathway metabolites? Do the changes in metabolites or pathway intermediates reflect increases or decreases in the levels of the enzymes that act on them?

The best understood aspect of the fission yeast transcriptional response to phosphate starvation is the central role of DNA-binding transcription factor Pho7, which recognizes a 12-nucleotide motif in the promoters of three phosphate acquisition genes (*pho1*, *pho84*, and *tgp1*) that are turned on during phosphate starvation (14–18). Time-resolved RNA-seq analyses of phosphate-starved wild-type and *pho7*∆ cells identified a larger Pho7 regulon, comprising 20 genes, upregulated by 9- to 2,500-fold, each of which has at least one, and as many as five, candidate Pho7 binding sites upstream of the TATA-box in the gene promoter (7). Members of the Pho7 regulon include the genes encoding: (i) aldehyde dehydrogenase Atd1 (upregulated by 100-fold during phosphate starvation) and (ii) glyceraldehyde-3-phosphate dehydrogenase Gpd3 (upregulated by 75-fold). In the case of Atd1, we suspect that its transcriptional upregulation is responsible for the accumulation of its reaction product betaine.

Phosphate starvation results in depletion of every glycolysis pathway intermediate between glucose and pyruvate, while every enzyme that converts glucose to pyruvate is transcriptionally upregulated, led by Gpd3 (by 75-fold) and enolase Eno102 (by 70-fold) (Table S1). Ample glucose is present in the ePMGT(–PO_4_) medium, and the starved cells are diluted into fresh ePMGT(–PO_4_) during the 24 h of starvation so that the *A*_600_ never exceeds 0.8 (i.e., they achieve G0 quiescence rather than stationary phase arrest). Five of the eight annotated fission yeast hexose transporters are transcriptionally upregulated during phosphate starvation, as follows: Ght2 (up 16-fold), Ght3 (up 30-fold), Ght5 (up 9-fold), Ght4 (up 2-fold), and Ght8 (up 2-fold) (1) (Table S1). Thus, it seems unlikely that a lack of available glucose causes the decrement in glycolytic intermediates. It is conceivable that fission yeast cells upregulate glycolytic enzymes and glycolytic flux to maintain a level of ATP production sufficient to survive for several days in the absence of external phosphate (and at a time when the Krebs cycle is seemingly not operating at full capacity). More direct assays of flux through the bioenergetics pathways would be needed to clarify the issue.

We note that Kim et al. (19) recently reported a metabolomics analysis of budding yeast *Saccharomyces cerevisiae* grown for 8 h in synthetic complete medium containing either 10 mM phosphate or 0 mM phosphate and thereby identified 31 metabolites that increased and 49 metabolites that decreased by greater than or equal to a twofold at this single time sampled during phosphate starvation. Of the metabolites that decreased in their study, five were standard amino acids. In our case, the ePMGT medium contained ample amounts of all 20 standard amino acids, which engendered a high signal for these compounds in the medium-only blank samples as well as the cellular samples, thereby obscuring the potential effects of phosphate starvation on fission yeast amino acid pools. For those metabolites that were detected at 8 h of starvation in budding yeast and at 4 or 8 h of starvation in fission yeast, there were many instances in which the direction of change was concordant. For example, citrate, methylthioadenosine, pyridoxal, trimethyllysine, and nucleosides uridine, xanthosine, and guanosine increased in both studies (see Fig. S1 for phosphate starvation effects on fission yeast nucleoside levels), whereas dATP, ATP, CDP, UDP, GMP, CMP, UMP, IMP, NAD^+^, NADP^+^, NaMN, ADP-ribose, orotate, acetyl-CoA, glucose/fructose-6-phosphate, glyceraldehyde-3-phosphate, glycerol-3-phosphate, 3-phosphoglycerate, phosphoenolpyruvate, ribose-5-phosphate, and AdoHcy were decreased. These findings suggest that highly divergent fungi manifest a similar metabolic response to phosphate deficiency. There were, however, several examples of divergent effects, to wit, (i) AdoMet increased by threefold after 8 h of phosphate starvation in budding yeast but was rapidly depleted in fission yeast, (ii) AMP declined by ninefold in budding yeast but increased modestly in fission yeast, (iii) cadaverine increased by ninefold in budding yeast but was unaffected in fission yeast, and (iv) citrulline declined by fivefold in budding yeast but was unaffected in fission yeast (Table S2).

In conclusion, the present study fortifies the case for fission yeast as an attractive model system to integrate multi-omics approaches to understand the temporal events underlying cellular phosphate homeostasis in a phosphate-challenged environment.

## MATERIALS AND METHODS

### Growth media

YES is a complex rich medium containing 3% glucose, 0.5% yeast extract, 1.31 mM adenine, 1.45 mM histidine, 1.71 mM leucine, 2.01 mM uracil, and 1.3 mM lysine (20). YES medium contains 2.2 mM phosphate derived from the yeast extract component of YES. ePMGT(+PO_4_) medium (enhanced Pombe Minimal Glutamate with Thiamine; containing 15.5 mM phosphate) is a rich synthetic medium customized by the addition of 20 amino acids to achieve the same concentrations contributed by the yeast extract present in YES medium (1). Thiamine (15 µM) is included in ePMGT to maintain repression of Pho4 acid phosphatase expression (1). The recipe for 1 L of ePMGT(+PO_4_) medium contains the following ingredients: potassium hydrogen phthalate (3.0 g), anhydrous sodium phosphate dibasic (1.66 g), anhydrous sodium phosphate monobasic (0.46 g), glucose (20 g), adenine (0.25 g), uracil (0.25 g), glutamic acid (3.75 g), histidine (0.25 g), lysine (0.25 g), leucine (0.25 g), thiamine (5 mg), 1,000× vitamins (1 mL), 10,000× minerals (0.1 mL), 50× salts (20 mL), and amino acid mix (2.7 g). The components of the vitamin, minerals, and salt stocks are as defined previously (20). The amino acid mix is composed of alanine (2.8 g), arginine (1.3 g), asparagine (0.5 g), aspartic acid (2.65 g), cysteine (0.10 g), glutamine (0.1 g), glutamic acid (4.70 g), glycine (1.50 g), histidine (0.65 g), isoleucine (1.5 g), leucine (2.0 g), lysine (2.3 g), methionine (0.4 g), phenylalanine (1.3 g), proline (1.0 g), serine (0.8 g), threonine (0.8 g), tryptophan (0.25 g), tyrosine (0.60 g), and valine (1.75 g). The pH is adjusted to 5.6 as needed by the addition of NaOH. Sodium phosphate salts are omitted from ePMGT(–PO_4_) medium.

### Phosphate starvation

Six independent fission yeast cultures were maintained in logarithmic growth at 30°C in YES medium for 24 h, at which time cells (from cultures at *A*_600_ of 0.4 to 0.8) were harvested by centrifugation, washed with water, and resuspended at *A*_600_ of ∼0.3 in ePMGT(–PO_4_) or at *A*_600_ of ∼0.1 in ePMGT(+PO_4_). The cultures were incubated at 30°C and diluted as needed to not exceed *A*_600_ of 0.8. Aliquots (3.15 *A*_600_ units) of cells grown for 4, 8, 12, or 24 h in ePMGT(–PO_4_) and of cells grown for 24 h in ePMGT(+PO_4_) were collected by vacuum filtration onto a 25 mm 0.2 µm nylon membrane (Millipore Cat No. GNWP02500), and the membranes were immediately submerged in 1.2 mL of cold extraction solvent containing acetonitrile:methanol:water (4:4:2) and 1.5 µM ^13^C/^15^N amino acid mix (Cambridge Isotopes). The samples were vortexed and subjected to three cycles of freezing on dry ice and thawing at –20°C. Five media “blank” controls (ePMGT ±PO_4_) were collected by filtration and processed in parallel.

### Phosphate replenishment

Six independent fission yeast cultures were maintained in logarithmic growth at 30°C in YES medium for 24 h, at which time cells (from cultures at *A*_600_ of 0.4 to 0.8) were harvested by centrifugation, washed with water, and resuspended at *A*_600_ of ∼0.04 in ePMGT(–PO_4_) or at *A*_600_ of ∼0.2 in ePMGT(+PO_4_). The cultures were incubated at 30°C and diluted to not exceed *A*_600_ of 0.8. After 24 h, (i) 3.15 *A*_600_ units of cells grown in ePMGT+PO_4_ were collected by filtration and quenched in extraction solvent as described above; (ii) 3.15 *A*_600_ units of cells grown in ePMGT–PO_4_ were collected by filtration and quenched in extraction solvent; and (iii) cells grown in ePMGT–PO_4_ medium were harvested, washed with ePMGT(+PO_4_), and resuspended at *A*_600_ of ~0.30 in ePMGT(+PO_4_) medium. The cultures were incubated at 30°C and diluted once to maintain log-phase growth. At the times specified (2, 4, 6, 8, and 12 h after phosphate replenishment), 3.15 *A*_600_ units of cells were collected by filtration and quenched in extraction solvent. Five media “blank” controls (ePMGT ±PO_4_) were collected by filtration and processed in parallel.

### Metabolomics analyses

For metabolomic profiling using liquid chromatography-mass spectrometry, dried extracts were resuspended in 60 µL of water for ion pair liquid chromatography separation and in 50 µL of 60:40 acetonitrile:water for hydrophilic interaction liquid chromatography (HILIC). Samples were vortexed, incubated on ice for 20 min, and clarified by centrifugation at 20,000 *g* for 20 min at 4°C. Ion pair LC-MS analysis was performed on a 6230 TOF mass spectrometer with dual JetStream source (Agilent Technologies) in negative ionization mode using a Waters XSelect HSS T3 column (150 × 2.1 mm, 3.5 µm particle size) and applying a gradient of solvent A (5 mM octylamine and 5 mM acetic acid in water) and solvent B (5 mM octylamine and 5 mM acetic acid in 90:10 methanol:water), with a post-column flow consisting of a blend of acetone:DMSO (90:10) at 300 µL/min. The analytical gradient was 0–3.5 min, 1% B; 4–15 min, 35% B; 20–22 min, 100% B; and 22–27 min, 1% B. Other LC parameters were as follows: flow rate 300 µL/min, column temperature 40°C, and injection volume 5 µL. MS parameters were as follows: gas temp: 250°C; gas flow: 9 L/min; nebulizer pressure: 35 psig; sheath gas temp: 250°C; sheath gas flow: 12 L/min; VCap: 3500 V; and fragmentor: 125 V. Data were acquired from 50 to 1,700 m/z with active reference mass correction (m/z: 119.0363 and 966.0007) infused through a second nebulizer according to the manufacturer’s instructions.

HILIC LC-MS analysis was performed on a 6545 Q-TOF mass spectrometer with dual JetStream source (Agilent Technologies) in positive ionization mode using a Waters Acquity UPLC BEH Amide column (150 × 2.1 mm, 1.7 µm particle size) and applying a gradient of solvent A (10 mM NH4 acetate, 10% acetonitrile, and 0.2% acetic acid) and solvent B (10 mM NH4 acetate, 90% acetonitrile, and 0.2% acetic acid). The analytical gradient was 0–9 min, 95% B; 9–13 min, 60% B; 13–14 min, 30% B; and 14.5–20 min, 95% B. Other LC parameters were as follows: flow rate 400 µL/min, column temperature 40°C, and injection volume 5 µL. MS parameters were as follows: gas temp: 300°C; gas flow: 10 L/min; nebulizer pressure: 35 psig; sheath gas temp: 350°C; sheath gas flow: 12 L/min; VCap: 4000 V; nozzle voltage: 1000 V; and fragmentor: 125 V. Data were acquired from 50 to 1,700 m/z with active reference mass correction (m/z: 121.0508 and 922.0097) infused through a second nebulizer according to the manufacturer’s instructions.

Peak identification and integration were done based on exact mass and retention time matched to commercial standards. Data analysis was performed with Skyline software (21) and plotted using RStudio; the peak areas for the biological replicates of individual metabolites are plotted in bar graph format in the figures. Note that the phosphate starvation and phosphate replenishment experiments were performed and analyzed by mass spectrometry at separate times. Because mass spectrometer performance can vary over time, causing the ion abundance observed for a given metabolite to differ between the starvation and repletion experiments, the y-axis scales for some of the metabolites may differ for the starvation and replenishment graphs.

## Data Availability

The raw LC/MS data in this study are available via the MassIVE database at https://massive.ucsd.edu/ProteoSAFe/dataset.jsp?task=11b4626b276246319fb412b8d669992f.

## SUPPLEMENTAL MATERIAL

Supplemental Material mbio.00241-25-s0001.pdf Fig. S1; captions to Tables S1 to S3.

Table S1 mbio.00241-25-s0002.xlsx Transcriptome profile as a function of time of phosphate starvation.

Table S2 mbio.00241-25-s0003.xlsx List of metabolites detected, with peaks areas for biological replicates.

Table S3 mbio.00241-25-s0004.xlsx List of metabolites reported in Figures 1-12, with peak areas for biological replicates.

ASM does not own the copyrights to Supplemental Material that may be linked to, or accessed through, an article. The authors have granted ASM a non-exclusive, world-wide license to publish the Supplemental Material files. Please contact the corresponding author directly for reuse.
