# Supplementary material for: Fission yeast metabolome dynamics during phosphate starvation and replenishment
Source: mBio. 2025 Feb 25;16(4):e00241-25. doi: 10.1128/mbio.00241-25 (PMC11980565; doi:10.1128/mbio.00241-25)
Supplement: Supplemental Material — Fig. S1; captions to Tables S1 to S3. [file mbio.00241-25-s0001.pdf]

Supplemental Material

Fission yeast metabolome dynamics during phosphate starvation and replenishment

Ana M. Sanchez, Aye K. Kyaw, Sara Nunes Violante, Angad Garg, Justin R. Cross, and Stewart Shuman

Figure S1

Table S1, S2, and S3 legends

Tables S1, S2, and S3 are provided as separate .xls files

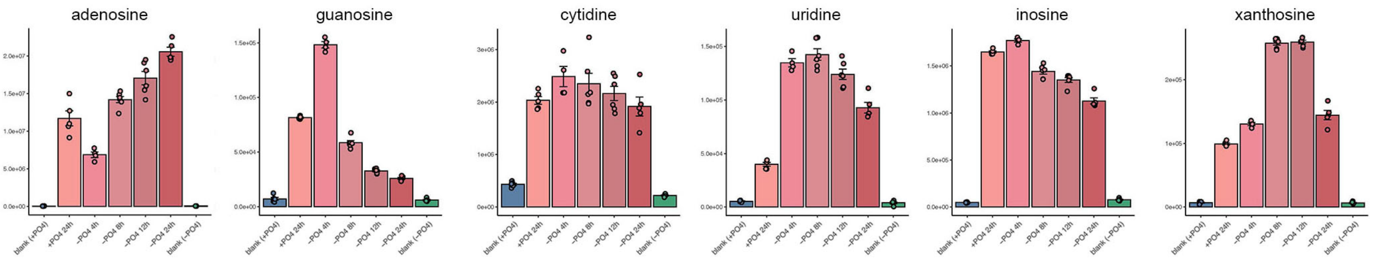

Figure S1. The bar graphs depict the levels of nucleosides adenosine, guanosine, cytidine, uridine, inosine, and xanthosine during phosphate starvation.

Table S1 legend.

RNA-seq analysis of transcriptome changes during 4, 8, 12, and 24 h of phosphate starvation.

List of protein-coding transcripts that were upregulated by ≥2-fold (page 24 UP) or downregulated by ≥2-fold (page 24 DOWN) at two or more sequential timepoints. The data, expressed as log2 fold change at each time versus the time 0 (phosphate-replete) control, are from Garg et al. (2023) Nucleic Acids Res 51: 3094-3115. PMCID: PMC10123115. Gene expression changes that are cited in the Results section of the present paper are highlighted in yellow shading in the Table.

Table S2 legend.

Standard-verified metabolites that were considered in the present study.

Metabolites surveyed as a function of starvation time (page Starvation) and as a function of recovery from starvation (page Recovery) are listed in column A. The method of detection is shown in column B. Peak areas for multiple biological replicates are compiled for each metabolite alongside medium-only “Blank” control values.

Table S3 legend.

Metabolites plotted in Figures 1 through 12.

Metabolites are listed in columns A and B according to their appearance in the bars graphs in Figures 1 though 12. Peak areas for multiple biological replicates in the starvation experiment (page Starvation_Metabolites_Figs1-12) and the recovery experiment (page Recovery_Metabolites_Figs1-12) are compiled for each metabolite alongside the medium-only “Blank” control values.
